# Application of commercial ultrafiltration membranes for protein purification by high performance countercurrent membrane purification (HPCMP)

**DOI:** 10.1002/btpr.88537

**Published:** 2026-07-07

**Authors:** Aylin Mohammadzadehmarandi, Nate Heller, Mohammadreza Ahmadimashhadi, Jon Petrone, Nicholas Marchand, Siavash Darvishmanesh, Ka Zhang, Stefano Menegatti, Andrew L. Zydney

**Affiliations:** ^1^ Department of Chemical Engineering The Pennsylvania State University University Park Pennsylvania USA; ^2^ Department of Biomedical Engineering The Pennsylvania State University University Park Pennsylvania USA; ^3^ Cytiva Marlborough Massachusetts USA; ^4^ Department of Chemical Engineering The North Carolina State University Raleigh North Carolina USA

**Keywords:** bioprocessing, bioseparations, diffusive transport, membranes, monoclonal antibody

## Abstract

High performance countercurrent membrane purification (HPCMP) has emerged as a promising diffusive separation strategy for the purification of recombinant proteins and monoclonal antibodies (mAbs). However, prior studies employed dialysis membranes, which are designed for diffusive separations but have not been validated for biopharmaceutical manufacturing. This study evaluates the ability of commercial ultrafiltration (UF) membranes, engineered for pressure‐driven filtration, to support highly selective diffusive separations. Scanning electron microscopy (SEM) analysis of the membrane cross‐sections revealed marked morphological differences between UF and dialysis membranes. Yet, transport studies using polydisperse dextrans and model proteins demonstrated that UF membranes can deliver exceptional diffusive separation selectivity. In particular, a 100 kDa polysulfone membrane (Cytiva) provided >150‐fold diffusive selectivity between bovine serum albumin (BSA) and immunoglobulin G (IgG). When utilized for mAb separation from a clarified CHO cell culture fluid, this membrane achieved >98% product yield, near‐complete removal of low molecular weight species, and significant reduction in both DNA and host cell protein (HCP) levels. These results demonstrate that commercial UF membranes that are validated for bioprocessing applications can be repurposed for HPCMP, enabling scalable, continuous downstream processing of biotherapeutics.

## 1 INTRODUCTION

The biopharmaceutical industry faces mounting pressure to reduce manufacturing costs of monoclonal antibody (mAb) therapeutics for both traditional applications (e.g., cancers and autoimmune disorders) and for the treatment of infectious disease.^1^, ^2^ For example, Lyke et al.^3^ recently demonstrated that mAbs that target the circumsporozoite protein in *Plasmodium falciparum*, the parasite that causes malaria, can provide a valuable option for protection/treatment of malaria, which currently effects more than 250 million people per year.^4^ However, these novel therapies can only realize their full public health potential if manufacturing costs are driven to levels that ensure accessibility in resource‐limited settings. In that context, the Gates Foundation recently established a “grand challenge” initiative awarding grants to develop end‐to‐end manufacturing platforms for anti‐malarial mAbs at costs below $10/g.^5^

One approach to significantly reduce the cost of mAb manufacturing is to replace expensive chromatographic processes—in particular the Protein A affinity capture—with more economical alternatives. Several groups have explored the use of selective precipitation for initial mAb capture/purification,^6^, ^7^, ^8^ and there has also been renewed interest in antibody crystallization^9^ and aqueous two‐phase systems for antibody extraction.^10^, ^11^ Membrane‐based separation methods offer significant advantages over traditional chromatographic techniques in terms of reduced operational costs and improved scalability.^12^, ^13^ Wang et al.^14^ recently described a fully membrane‐based downstream process that incorporates a series of membrane adsorbers for purifying multiple mAb products.

Recent work by Mohammadzadehmarandi et al.^15^ demonstrated that High Performance Countercurrent Membrane Purification (HPCMP) can significantly reduce host cell protein (HCP) levels in processing a clarified cell culture fluid (CCF) containing a mAb. HPCMP exploits differences in diffusive transport rates across semi‐permeable membranes for mAb purification, with low molecular weight HCP removed by diffusion into an appropriate draw solution. Subsequent work by Minervini et al.^16^ demonstrated that HPCMP can be integrated with single pass tangential flow filtration (SPTFF) into an intensified process for initial mAb purification. However, these studies relied on prototype dialysis membranes that are neither commercially available nor validated for GMP manufacturing. Previous studies of HPCMP for separation of model proteins (bovine serum albumin and myoglobin) utilized hemodialysis membranes (Purema™ H) that were developed for clinical applications and lack bioprocessing validation.^17^

This study addresses these limitations by systematically evaluating commercial hollow fiber UF membranes for mAb purification by HPCMP. Although these commercial membranes were designed for convective transport, the size‐selective pores should also provide at least some diffusive selectivity. Through a combination of dextran sieving characterization, model protein systems, and CCF purification studies, we identified and validated a commercial polysulfone (PS) membrane capable of achieving highly selective mAb purification, thereby facilitating industrial adoption of HPCMP technology.

## 2 MATERIALS AND METHODS

### 2.1 Hollow fiber membranes

Experiments were performed using two types of membranes: commercially available hollow fiber UF membranes (obtained from Cytiva and Repligen) and dialysis membrane modules. The key properties of these membranes/modules are summarized in Table 1. The 100 kDa molecular weight cutoff UF membrane from Cytiva is made of polysulfone (PS) while the UF membranes from Repligen are primarily a modified (hydrophilized) polyethersulfone (mPES). The dialysis membranes (bottom two entries in Table 1) are both polymer blends and have not previously been categorized with a specific molecular weight cutoff, but they are designed to retain serum albumin which has a MW = 67 kDa.

**TABLE 1:** Properties of hollow fiber membrane UF modules (first four entries) and dialyzer modules (bottom two entries).

Manufacturer	Module name	Polymer	Membrane area	Fiber inner diameter	Fiber length	Nominal MWCO (kDa)
Cytiva	MidGee	PS	26 ${\mathrm{cm}}^{2}$	500 μm	23 cm	100
Repligen	MidiKros	PS	190 ${\mathrm{cm}}^{2}$	500 μm	20 cm	50
Repligen	MidiKros	mPES	115 ${\mathrm{cm}}^{2}$	500 μm	20 cm	50
Repligen	MidiKros	mPES	115 ${\mathrm{cm}}^{2}$	500 μm	20 cm	70
Baxter	Theranova 400	Polyarylethersulfone and PVP blend	1.7 $m^{2}$	190 μm	23.6 cm	–
3M	Prototype	PES and PVP blend	40 ${\mathrm{cm}}^{2}$	200 μm	23 cm	–

The polysulfone (PS) membranes were soaked overnight in 20% isopropyl alcohol (IPA) to facilitate wetting of the pore space since these membranes are provided dry (without glycerin as a pore humectant). The other membranes were used as is. The modules were initially flushed with at least 50 L/m^2^ DI water, ensuring removal of any air bubbles, with the membrane permeability ($L_{p}$) evaluated from data for the filtrate flux ($J_{V}$), equal to the volumetric filtrate flow rate divided by the membrane area, as a function of the mean transmembrane pressure (TMP) using Equation (1):

(1) $$L_{p}=\frac{J_{V}}{\mathrm{TMP}}$$

The TMP was evaluated as the difference between the average pressure in the fiber lumens (average of feed inlet and retentate outlet) and the average pressure in the shell region, with all pressures measured using Ashcroft® digital pressure gauges placed immediately before and after the hollow fiber module. Only modules with permeabilities within the manufacturer's specified range were used for subsequent experiments.

Additional characterization of limited membranes was performed by Scanning Electron Microscopy (SEM) using a ThermoFisher Scientific Axia ChemiSEM. Hollow‐fiber modules were cut open with a fresh single‐edge razor blade, individual fibers were removed, mounted on aluminum stubs using conductive carbon tape, and sputter‐coated with a thin Au/Pd layer (~5–7 nm). Imaging of individual fibers was conducted under high vacuum using secondary electron detection at an accelerating voltage of 5 kV and a working distance of ~10 mm to visualize fiber morphology.

### 2.2 Dextran challenge

The diffusive properties of the membranes were evaluated using a dextran challenge consisting of a “cocktail” of several different MW dextrans: 0.2 g/L of Dextran T10 (9–11 kDa), 0.4 g/L of Dextran T40 (35–45 kDa), 0.8 g/L of Dextran T70 (70 kDa), and 1.8 g/L of Dextran T150 (150 kDa), obtained from MilliporeSigma (St. Louis, MO) and TCI Chemical (Portland, OR). The higher concentrations of the larger MW dextrans were used to obtain more accurate data for the concentrations of these highly retained species in the dilute draw solution. Dextrans were dissolved in a freshly made 1x PBS solution prepared by dilution of a 10x PBS stock solution (Thermo Fisher Scientific, Waltham, MA) with deionized (DI) water (Millipore Direct‐Q water purification system, Burlington, MA); the draw solution was PBS without any added dextrans. All solutions were pre‐filtered through 0.22 μm polyvinylidene fluoride (PVDF) Durapore membranes (MilliporeSigma) prior to use.

The hollow fiber modules were mounted vertically with the feed solution introduced to the fiber lumens at the bottom of the module and the draw solution introduced into the shell side through the port at the far end of the module in a countercurrent configuration (Figure 1). Feed and draw solution flow rates were regulated using two Masterflex L/S peristaltic pumps (Cole‐Parmer, Vernon Hills, IL) equipped with platinum‐cured silicone tubing; the pump on the draw solution was fitted with two identical pump heads to maintain the same draw solution flow rate into and out of the module. This ensured that the modules were operated with minimal convective (pressure‐driven) filtration. Each pump was calibrated prior to the experiments by timed collection using a digital balance. Experiments were performed with the same residence time ($t_{R}$) in the different modules, evaluated from Equation (2):

(2) $$t_{R}=\frac{V}{q_{F}}$$

where *V* is the total internal volume of the hollow fiber lumens and $q_{F}$ is the volumetric feed flow rate. Operation at the same residence time provides equivalent times for diffusive solute removal in all modules; future studies will be required to compare operation under different (optimized) experimental conditions for each module based on differences in diffusive transport rates. Note that a residence time of 65 min corresponds to a feed flow rate of 0.005 mL/min for the Cytiva 100 kDa module; the feed flow rates for the other modules were adjusted based on Equation (2) and the values of the module volumes determined from the fiber geometries in Table 1.

**FIGURE 1:**
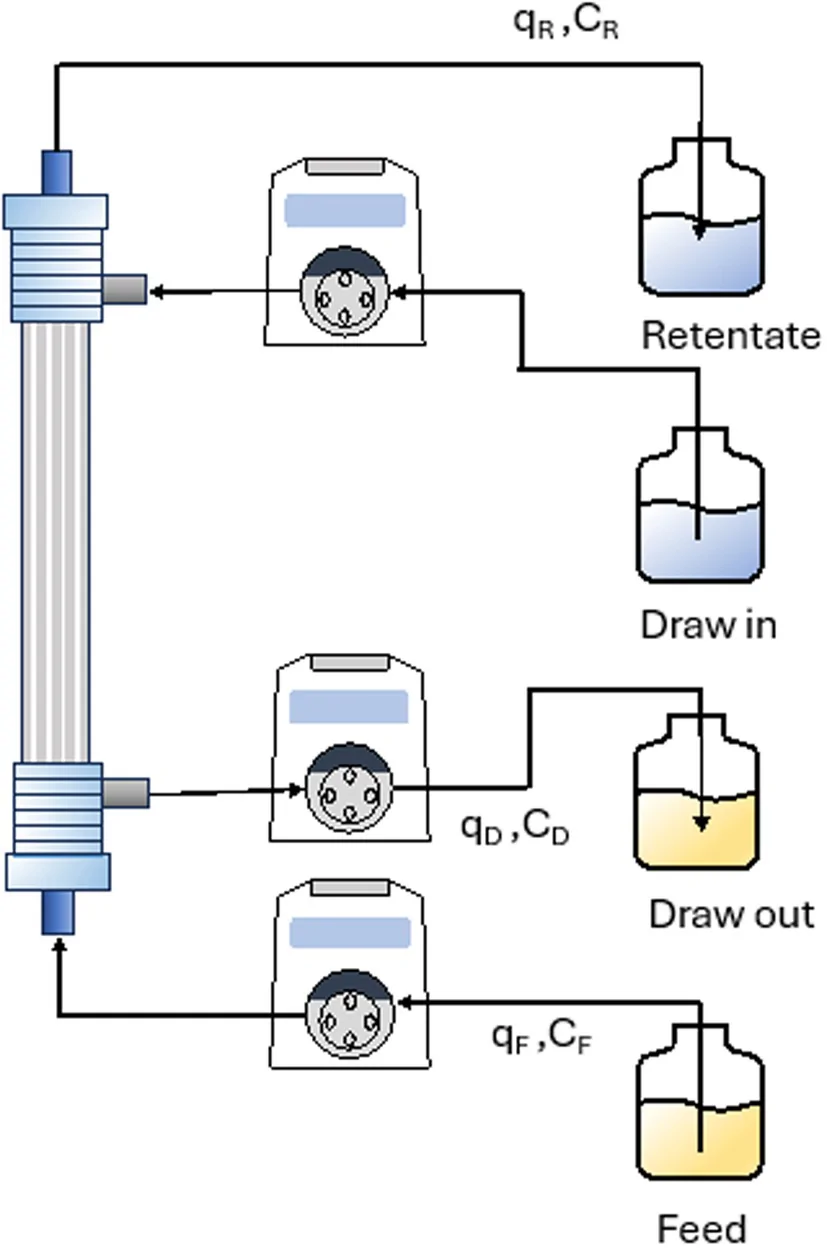
Schematic of continuous countercurrent membrane purification system.

Dextran concentrations were evaluated using a high‐performance liquid chromatography (HPLC) system (Agilent 1260 Infinity II, Agilent Technologies, Santa Clara, CA) equipped with a refractive index (RI) detector. To enable effective separation of dextran molecules across the full range of molecular weight, Ultrahydrogel 500 and Ultrahydrogel 120 (Waters Corporation, Milford, MA) columns were connected in series, with a guard column positioned immediately upstream of the Ultrahydrogel 500. Columns were cleaned with 0.1 N NaOH and replaced periodically; small shifts in SEC results reflect differences between columns. The HPLC was operated at a flow rate of 0.6 mL/min, using a column temperature of 30°C. Sample injections were performed at intervals ranging from 60 to 90 min. Calibration was carried out before each set of dextran samples using polyethylene oxide (PEO) molecular weight standards (EasiVial, Agilent Technologies), with a calibration curve generated by plotting the logarithm of the PEO molecular weight as a function of retention time (with R^2^ > 0.99 by simple linear regression).

Fractional dextran removal (R_D_) was calculated as follows:

(3) $$R_{D}=\frac{q_{D}C_{D}}{q_{F}C_{F}}$$

where *q*_D_ and *C*_D_ are the draw solution flow rate and dextran concentration, and the subscripts “*F*” and “*D*” denote the flow rate and concentrations evaluated in the feed and draw solution, respectively. All experiments used *q*_D_/*q*_F_ = 4 to provide a strong concentration driving force without excessive dilution; the extent of impurity removal can be increased by increasing *q*_D_/*q*_F_, but this would require the use of greater buffer volumes. The choice of *q*_D_/*q*_F_ = 4 was guided by theoretical calculations presented in Yehl and Zydney^18^ showing that the use of higher draw solution flow rates provides minimal additional impurity removal. Mass balances were typically closed within ±5% based on simultaneous sampling of draw (buffer) and retentate (product) exits.

### 2.3 Model proteins

Additional characterization of the hollow fiber membranes was performed using bovine serum albumin (BSA, MW ~ 67 kDa) and human serum immunoglobulin G (IgG, MW ~ 150 kDa) as model proteins. BSA was sourced from MilliporeSigma (St. Louis, MO), while IgG was obtained from Nova Biologics (Oceanside, CA). Both BSA and IgG were dissolved in PBS and then prefiltered through the 0.22 μm Durapore PVDF membranes. BSA and IgG concentrations were evaluated by UV absorbance at 280 nm using an Infinite® m200 Pro microplate reader (Tecan Trading AG, Switzerland).

### 2.4 Clarified cell culture fluid

Harvested cell culture fluid (CCF) was produced by culturing Chinese Hamster Ovary (CHO) cells in a 3‐L stirred tank bioreactor as described by Zhang et al.^19^ The cells were engineered to produce the NIST mAb (reference material RM 8671), which is a humanized IgG1κ with a MW ≈ 150 kDa and pI ≈ 7.4.^20^ The initial mAb titer was 0.7 g/L based on Protein A chromatography performed with a POROS A column (Thermo Fisher Scientific, Waltham, MA). The CCF was then concentrated by SPTFF using a Theranova 400 (Baxter, Deerfield, Il) hollow fiber polyethersulfone membrane at feed and permeate flow rates of 60 and 57 mL/min, respectively, corresponding to a 20‐fold concentration factor, following the procedures described by Minervini et al.^16^ SPTFF was performed in total recycle mode to demonstrate stable operation over 24 h given the limited volume of HCCF. The collected retentate and the holdup volume at the end of the SPTFF was frozen, stored at −20°C, and then gently thawed before use as a feed for HPCMP, which was performed with 150 mM PBS as the draw solution to approximately match the conductivity of the feed.

The distribution of HCP and low MW impurities in the CCF was characterized by size exclusion chromatography (SEC) using a Vanquish UHPLC equipped with a BioBasic SEC300 column (300 mm × 7.8 mm) from Thermo Fisher Scientific. Detection was by UV absorbance at 280 nm. The UHPLC was operated at a constant flow rate of 1 mL/min, with the column temperature maintained at 30°C. Samples were injected every 30 min using 150 mM PBS as the mobile phase.

DNA concentrations were evaluated using the Qubit® dsDNA BR (Broad‐Range) Assay Kit along with the Qubit® dsDNA HS Assay Kit (both from Thermo Fisher Scientific) with limits of quantification of 100 and 10 ng/mL, respectively. Total HCP concentrations were determined using a CHO HCP ELISA kit (Cygnus Technologies, Southport, NC).

## 3 RESULTS AND ANALYSIS

### 3.1 Membrane characterization

The different morphologies of the UF and dialysis membranes were examined by scanning electron microscopy. Cross‐sectional SEM images of the Cytiva 100 kDa UF membrane and Baxter Theranova 400 dialyzer membrane revealed striking morphological differences (Figure 2). The Cytiva UF membrane exhibited an inner diameter of 400–600 μm, in agreement with the manufacturer's reported value of 0.5 mm (500 μm), while the overall membrane wall thickness of approximately 200 μm provides the mechanical stability needed for UF applications at high transmembrane pressures. The membrane appears relatively homogeneous at this magnification, although it has a dense size‐selective skin at the inner (lumen) surface of the fiber. The morphologies of the other UF membranes were similar to that of the Cytiva 100 kDa membrane.

**FIGURE 2:**
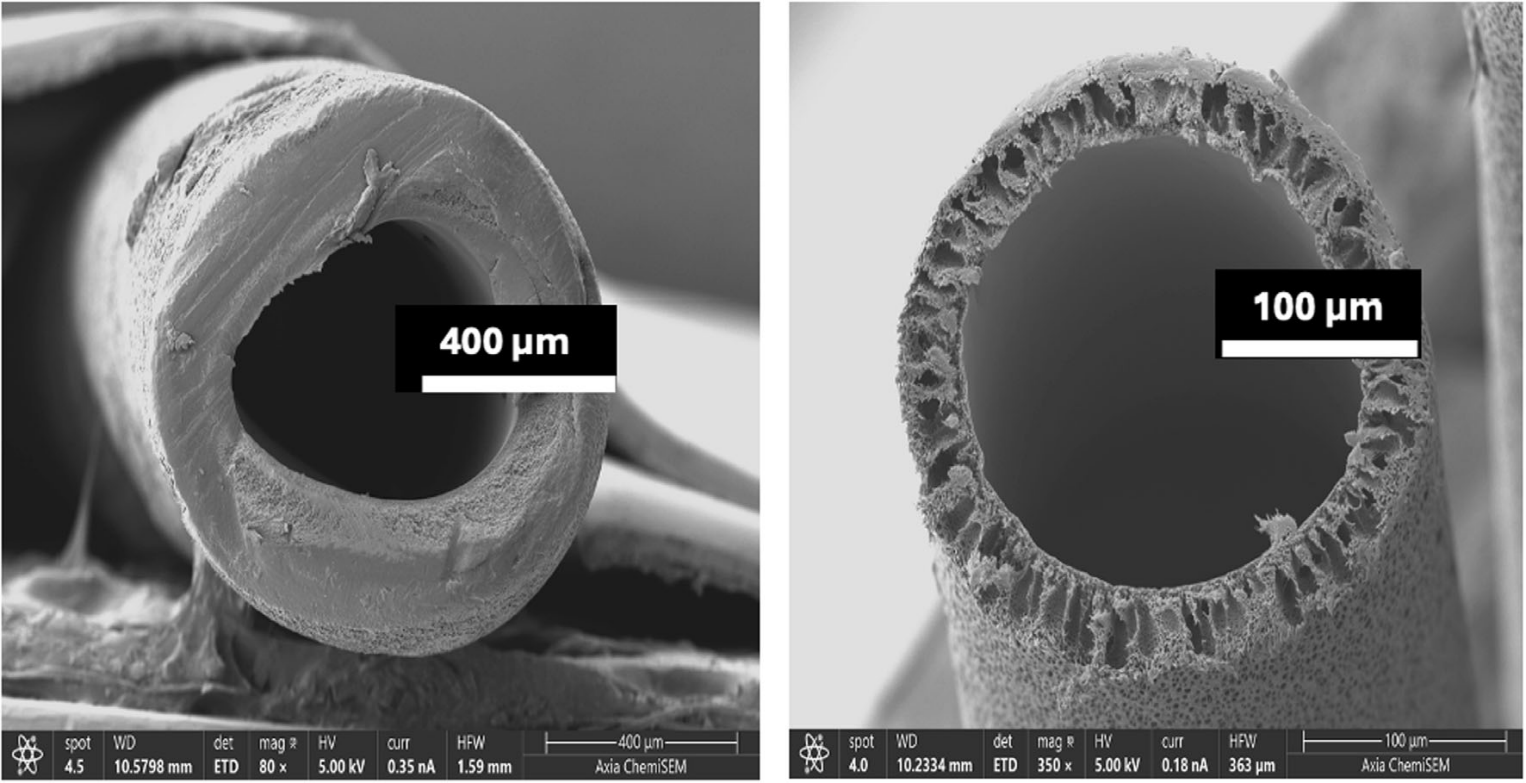
SEM images of the cross‐section of individual hollow fibers obtained from the Cytiva 100 kDa UF membrane (left) and the Baxter Theranova 400 dialyzer (right). Scale bars for the SEM images (shown in lower right) are 400 μm for the Cytiva 100 kDa UF membrane and 100 μm for the Baxter Theranova 400 dialyzer.

In contrast, the Baxter dialyzer membrane features an inner diameter of only 200 μm (approximately one‐half that of the Cytiva membrane) and a markedly thinner wall of approximately 35 μm. The dialyzer membrane exhibited a distinctly microporous substructure with large voids traversing the thickness, a design optimized for the high diffusion rates of metabolites essential in hemodialysis.

### 3.2 Dextran HPCMP

The different hollow fiber UF membranes were challenged with the dextran mixture, with samples obtained from the feed, retentate, and draw solutions for analysis by SEC. Typical results for the Baxter Theranova 400 dialysis membrane (top panel) and the Repligen 50 kDa UF membrane are shown in Figure 3. In both cases, data were obtained using *q*_D_/*q*_F_ = 4, with the feed flow rates adjusted to give residence times of approximately 65 min based on Equation (2). Previous studies have shown that residence times of at least 60 min are required to provide significant removal of host cell proteins by diffusive separations using dialysis membranes.^15^ Samples were analyzed after 10 h of operation to ensure steady‐state behavior. The feed appears bimodal, with dextrans eluting between 17 and 29 min. The larger dextrans (which elute at shorter times) are completely absent in the draw solution since they show negligible diffusive transport across either of the membranes. The Theranova 400 shows nearly complete removal of dextrans with *t* > 25.5 min (this is shown more clearly in Figure 4). Although the MidiKros 50 kDa membrane was designed for pressure‐driven filtration, this UF membrane provides significant diffusive removal of the smaller dextrans. However, in contrast to the Theranova 400, there is a small degree of retention even for dextrans with *t* > 27 min.

**FIGURE 3:**
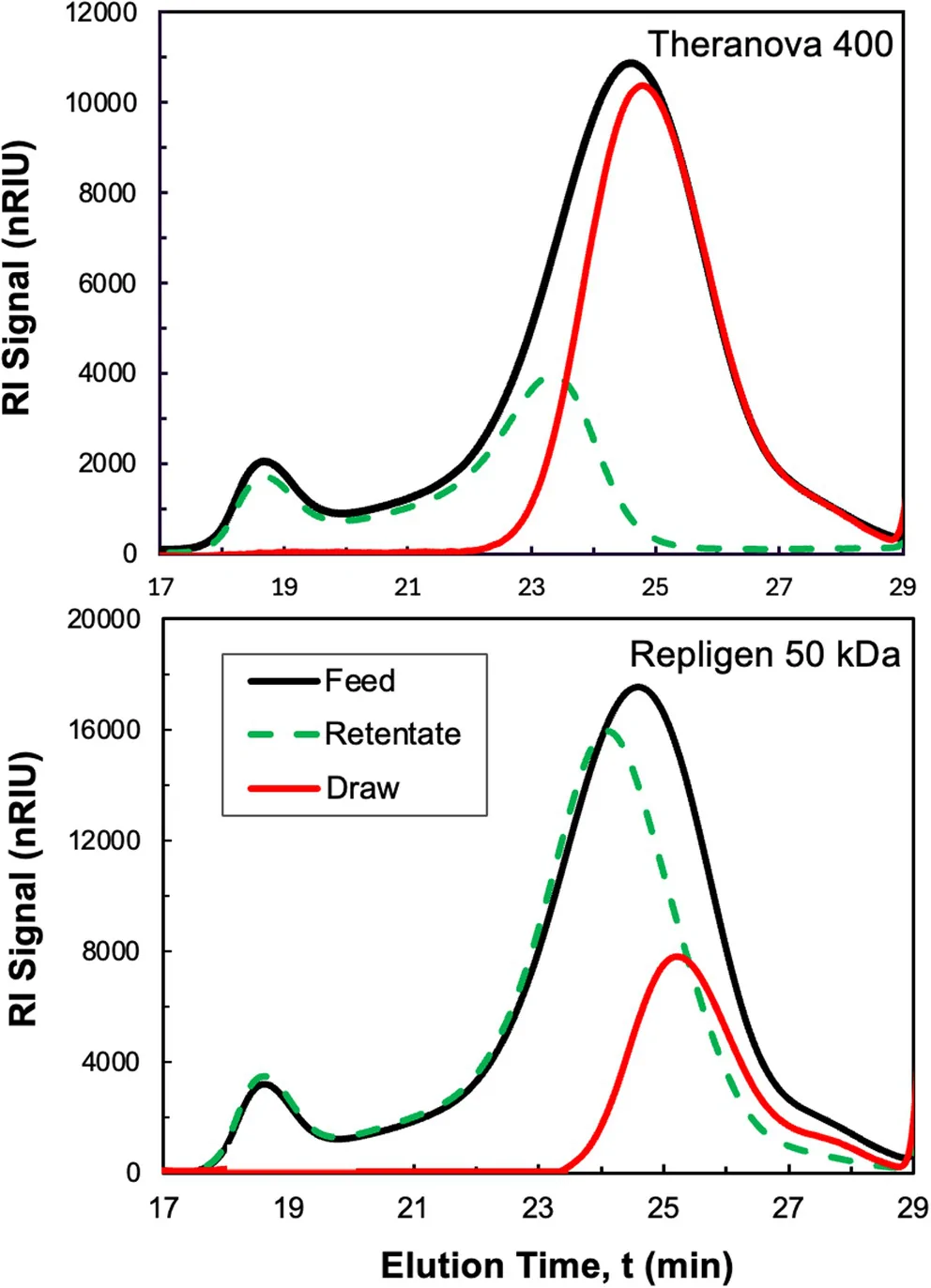
SEC chromatograms for samples of the feed, retentate, and draw solutions obtained from the Baxter Theranova 400 dialyzer (top) and Repligen 50 kDa UF membrane. RI signal for the draw solution has been multiplied by a factor of four to account for the dilution effect for *q*_D_/*q*_F_ = 4. The observed differences in absorbance values are likely attributable to variability in the SEC columns used for the two experiments.

**FIGURE 4:**
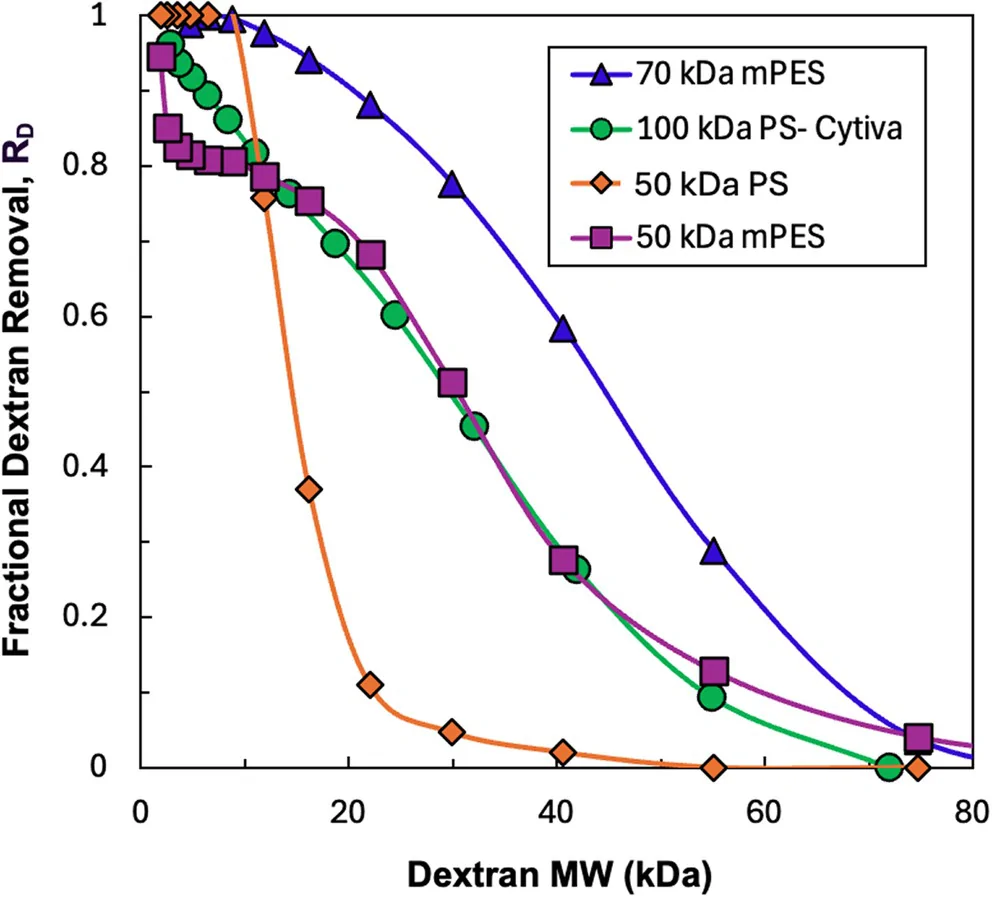
Fractional dextran removal as a function of the dextran MW for the Cytiva 100 kDa and Repligen 50 kDa PS, 50 kDa mPES, and 70 kDa mPES membranes.

The data in Figure 3 were used to evaluate the fractional dextran removal (R_D_) using Equation (3), with the dextran concentrations in the draw and feed samples evaluated as a function of the dextran MW using thin “slices” of the chromatograms corresponding to approximately 1 min increments in the elution time. Results are shown in Figure 4 for several of the UF membranes, with R_D_ plotted as a function of the dextran MW, which was determined from a calibration curve for the elution time as a function of MW constructed using SEC data for narrow PEO standards covering the full range of MW. Mass balance closure was typically within ±5% based on samples obtained from both the draw and retentate exit.

The Repligen 50 kDa PS membrane provided the most restrictive performance, showing minimal removal of dextrans >50 kDa, while also exhibiting a steep sieving curve that would enable selective separation of smaller solutes. The Cytiva 100 kDa PS and the Repligen 50 kDa mPES membranes exhibited nearly superimposable dextran removal profiles (with minor differences for dextrans <10 kDa), despite the different values of nominal molecular weight cut‐offs provided by the manufacturers (which were evaluated under pressure‐driven conditions using proprietary challenge tests that likely differ between manufacturers). The very similar diffusive profiles for these membranes suggest that they have similar pore size distributions, although dextran partitioning into the pores may also be affected by differences in membrane chemistry (PS vs. mPES). The Repligen 70 kDa mPES membrane demonstrated the highest dextran removal across all molecular weights, suggesting a larger effective pore size than the other tested membranes.

The fractional dextran removal curve for the Cytiva 100 kDa UF membrane is compared with that of the Baxter Theranova 400 and 3M dialyzers in Figure 5. These specific membranes were selected for final comparison based on the insights gained from parallel protein removal studies detailed in the next section. The objective was to compare the diffusive characteristics of a UF membrane, which is designed for pressure‐driven convective transport, with that of a dialyzer, which is designed primarily for diffusive transport. The feed flow rate for the large‐scale Baxter dialyzer was 0.5 mL/min to achieve the same residence time as used in the small‐scale modules. Although all three membranes exhibited qualitatively similar R_D_ profiles, the dialyzers displayed steeper removal curves, consistent with greater selectivity. This behavior is attributable to the much thicker substructure in the UF membrane (seen in the SEM image in Figure 2), which provides a significant additional resistance to diffusion but with minimal diffusive selectivity due to the very large pores in this region of the hollow fiber. Note that the substructure has essentially no effect on the convective selectivity, which is determined almost entirely by the properties of the size‐selective skin layer. The Cytiva 100 kDa UF membrane achieved *R*_D_ = 0.27 for the 40 kDa dextran, compared to only 0.15 and 0.09 for the Baxter Theranova 400 and 3M dialyzers, respectively. The limited removal of albumin‐sized dextrans by the dialyzers is consistent with the clinical requirement for minimal levels of albumin loss during hemodialysis.

**FIGURE 5:**
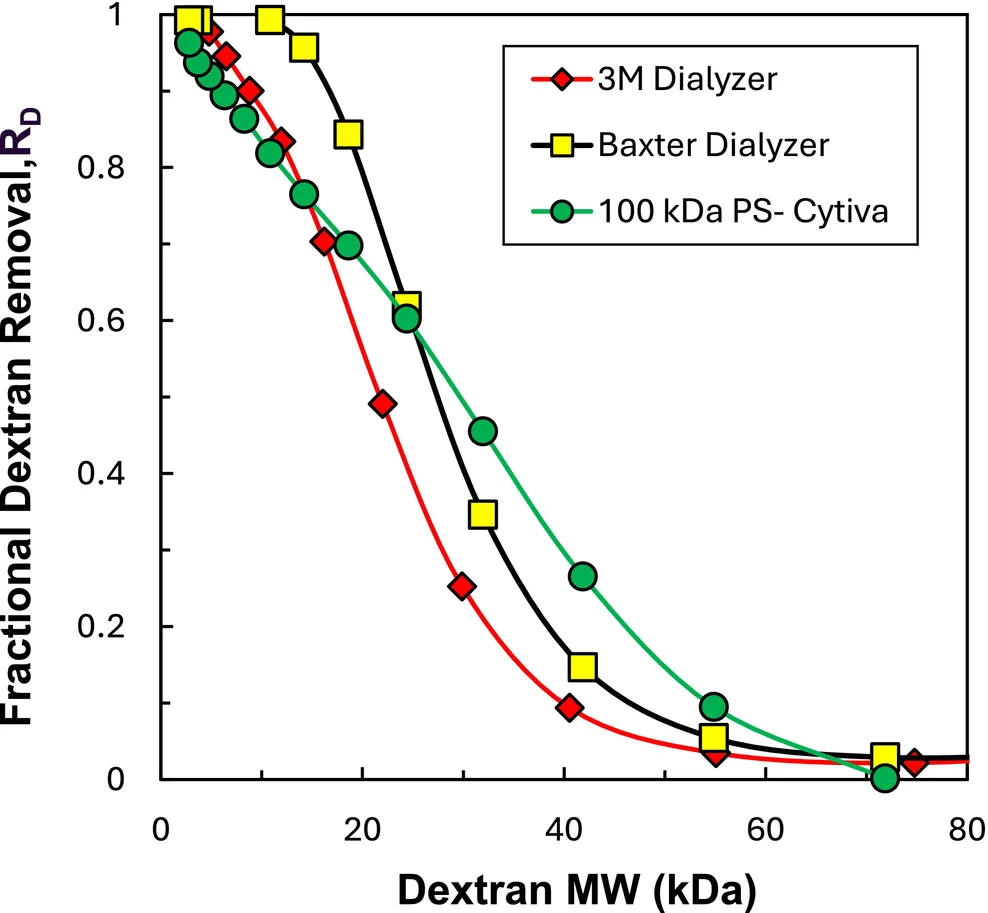
Comparison of the fractional dextran removal curves for the Baxter Theranova and 3M dialyzer membranes with that of the Cytiva 100 kDa UF membrane.

### 3.3 Model protein HPCMP and selectivity analysis

Further characterization of the membranes was conducted using BSA and serum (polyclonal) IgG as model proteins, representing HCP and mAb, respectively. The HPCMP data were obtained with separate solutions of the two proteins so that the fractional protein removal could be evaluated directly by UV absorbance measurements for the feed and draw solution. All experiments were performed with *q*_D_/*q*_F_ = 4, with the exit retentate flow rates ($q_{R}$) within ±5% of the feed flow rate ($q_{F}$). PBS was used as the draw solution. In this case, the removal rates were calculated based on the concentrations in the feed and retentate exit (which would be the product solution for an HPCMP process) using Equation (4):

(4) $$R_{F}=1-\frac{q_{R}C_{R}}{q_{F}C_{F}}$$

Results are summarized in Table 2 for the four UF membranes examined in Figure 4 along with literature data for the prototype 3M dialyzer from Mohammadzadehmarandi et al.^15^ The measured values of the protein removal were highly reproducible, with variation between multiple repeat measurements of less than ±10%. All 5 membranes provide reasonable levels of BSA removal; the high level of protein removal by the Repligen 50 kDa PS membrane is likely due to a combination of protein diffusion across the membrane and protein adsorption to the more hydrophobic polysulfone surface. However, only the 3M dialyzer and the Cytiva 100 kDa membrane achieved the high IgG retention (small *R*_F_) required for mAb purification by HPCMP. The very low level of IgG removal by the Cytiva 100 kDa membrane, which was confirmed by the absence of any measurable IgG concentration in multiple draw samples, is notable given its dextran sieving profile in Figures 4 and 5. This apparent discrepancy likely reflects substantial IgG fouling on the hydrophobic polysulfone surface, which would create an additional transport barrier either on or within the membrane pores that effectively reduces the rate of IgG diffusion. Although there may be significant differences in performance between the polyclonal IgG and specific monoclonal antibodies, the high diffusive selectivity of the Cytiva 100 kDa membrane for separating BSA and IgG motivates the use of this UF membrane for processing HCCF; this is examined in Section 3.4.

**TABLE 2:** Summary of results for BSA and IgG using the different UF membranes (first four entries) and the 3M dialyzer membrane (last entry).

Membrane	Feed flow rate, $q_{F}$ (mL/min)	BSA Removal, $R_{F,\mathrm{BSA}}$	IgG Removal, $R_{F,\mathrm{IgG}}$	Selectivity, $R_{F,\mathrm{BSA}}$/$R_{F,\mathrm{IgG}}$	Selectivity, $k_{o,\mathrm{BSA}}$/$k_{o,\mathrm{IgG}}$
Cytiva 100 kDa	0.005	37 ± 2	0.20 ± 0.04	190 ± 38	250 ± 52
Repligen 50 kDa PS	0.005	59 ± 4	13 ± 3	4.5 ± 1.1	7.1 ± 2.2
Repligen 50 kDa mPES	0.07	17 ± 1	2.0 ± 0.3	8.5 ± 1.4	9.1 ± 1.8
Repligen 70 kDa mPES	0.07	24 ± 2	5.6 ± 0.6	4.3 ± 0.6	4.9 ± 0.9
3M Dialyzer	0.04 mL/min	20 ± 2	0.44 ± 0.08	45 ± 9	55 ± 12

*Note*: Data for 3M dialyzer were taken from Mohammadzadehmarandi et al.^15^.

Since the fractional protein removal depends on the draw and feed solution flow rates, as well as the membrane area, the experimental data in Table 2 were used to evaluate the overall mass transfer coefficients for both BSA and IgG based on classical models for countercurrent contacting^17^, ^21^:

(5) $$\frac{C_{R}}{C_{F}}=\frac{\left(\alpha -1\right)\exp\left(-\beta \right)}{\alpha -\exp\left(-\beta \right)}$$

where *α = q*_*D*_ */q*_*F*_ and

(6) $$\beta =\frac{k_{o}A}{q_{F}}\left(1-\frac{1}{\alpha }\right)$$

The final column in Table 2 shows the calculated selectivity based on the ratio of the overall mass transfer coefficients for BSA and IgG determined using Equations (5) and (6). The general trends in the mass transfer coefficient selectivity are identical to those for *R*_F,BSA_/*R*_F,IgG_, with the highest selectivity obtained for the Cytiva 100 kDa PS UF membrane. Note that the mass transfer coefficient selectivity provides a measure of the maximum value of *R*_F,BSA_/*R*_F,IgG_ (obtained at α ➔ ∞ and very small q_F_).

For a homogeneous membrane, the mass transfer coefficient is directly related to the effective solute diffusivity (D_eff_):

(7) $$k_{o}=\frac{\epsilon D_{\mathrm{eff}}}{\delta \tau }$$

where ϵ is the membrane porosity, τ is the membrane tortuosity, and δ is the membrane thickness. The observed selectivity between BSA and IgG is thus due in part to the difference in the bulk diffusion coefficients of the proteins. The diffusion coefficient for IgG is *D*_o_ = 3.9 × 10^−11^ m^2^/s^22^ while that for BSA is *D*_o_ = 6.0 × 10^−11^ m^2^/s,^23^ which would provide a mass transfer coefficient selectivity of only 1.54, which is well below the values reported in Table 2, clearly demonstrating the importance of the hindrance to diffusion provided by the porous membrane.

The effective diffusion coefficient in a cylindrical pore can be evaluated using the polynomial fit presented by Dechadilok and Deen^24^:

(8) $$\frac{D_{\mathrm{eff}}}{D_{o}}=\begin{array}{l} 1+\frac{9}{8}\ln\left(\lambda \right)-1.56034\lambda +0.528155\lambda^{2}+1.91521\lambda^{3}-2.81903\lambda^{4}\\ +\;0.270788\lambda^{5}+1.10115\lambda^{6}-0.435933\lambda^{7} \end{array}$$

where *λ* is the ratio of the solute to pore radius; Equation (8) is accurate to within 2% for limiting expressions at both small and large λ. Note that Equation (8) neglects any electrostatic effects on solute partitioning or hindered diffusion. This assumption should be reasonable given the relatively high ionic strength (150 mM) of the CCF and PBS, which will effectively shield the electrostatic interactions. The data for the 3M dialyzer yielded k_o_ = 7.3 × 10^−10^ m/s for the IgG, which gives *D*_eff_/*D*_o_ = 0.0026 using ε = 0.5, *τ* = 2, and δ = 35 μm; the thickness was evaluated directly from the SEM image in Figure 2 while the porosity and tortuosity were reasonable estimates for dialysis membranes. This corresponds to λ = 0.81 based on Equation (8). We can estimate the expected mass transfer selectivity between BSA and IgG from the ratio of the size of the two proteins (which should scale with the diffusivities based on the Stokes‐Einstein equation). This gives *λ* = 0.53 for BSA with *D*_eff_/*D*_o_ = 0.035, yielding a mass transfer coefficient selectivity of 33. This is somewhat below the value reported in Table 2, although this discrepancy is not unexpected given the simplifications in the analysis.

The analysis of the mass transfer coefficient selectivity for the UF membranes is more complicated since these membranes have a thin, highly selective skin layer and a much thicker microporous support that provides minimal selectivity (beyond the inherent selectivity arising from the differences in the diffusivities of BSA and IgG). The overall mass transfer coefficient for IgG for the Cytiva 100 kDa membrane was *k*_o_ = 6.2 × 10^−11^ m/s, more than an order of magnitude smaller than the value for the 3M dialyzer even though the selective skin of the Cytiva hollow fiber is probably less than 1 μm in thickness. This suggests that the hindrance to IgG transport in the pores of the Cytiva membrane is very high, leading to a very small value of *D*_eff_/*D*_o_ in the size‐selective skin.

### 3.4 mAb purification from a clarified CHO cell culture fluid by HPCMP

The initial mAb titer of only 0.7 g/L in the CCF necessitated preconcentration prior to HPCMP. SPTFF was performed using a Theranova 400 hollow fiber module (surface area of 1.8 m^2^, fiber inner diameter of 190 μm, priming volume of 93 mL) at a feed flow rate of 60 mL/min, based on results from Minervini et al.^15^ SPTFF was performed in total recycle mode to demonstrate stable performance over 26 h of continuous operation, with the transmembrane pressure remaining below 20 kPa throughout the experiment. The retentate product at the end of the SPTFF process was then collected for subsequent analysis and purification by HPCMP. Analytical Protein A chromatography confirmed complete mAb retention (99% recovery based on final retentate volume and concentration), with the concentrated solution having a mAb concentration of 8 g/L mAb (after dilution by the buffer flush used to recover mAb from the hollow fiber module).

While designed for preconcentration prior to HPCMP, the Theranova membrane also provided a significant removal of low MW species, as evidenced by the SEC analysis in Figure 6, which compares the initial feed and the retentate product after SPTFF. The mAb peak eluted at 15 min (confirmed by independent measurements with the purified mAb). The SEC absorbance data for the retentate product was normalized by the mAb peak to highlight the removal of low MW species (eluting at *t* > 20 min). The HCP concentration, measured by CHO HCP ELISA, decreased from 224,000 ppm in the feed to 76,000 ppm in the retentate, representing a 66% reduction. The total A_280_ signal from low‐MW components (integrated from *t* = 20–35 min using area under the curve principles) decreased >90%, consistent with removal of cell culture media components including amino acids and vitamins.

**FIGURE 6:**
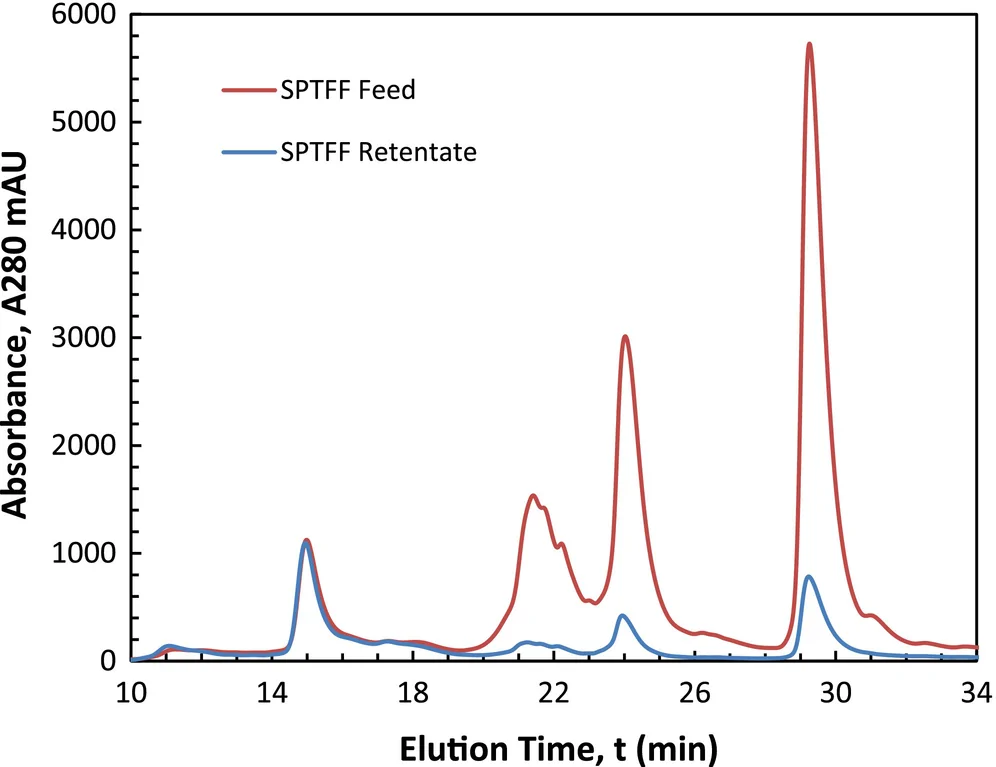
SEC chromatograms: The red trace represents the initial feed, while the blue trace corresponds to the retentate product obtained through SPTFF. The absorbance values for the retentate have been normalized by the mAb peak to highlight the removal of low MW components.

The DNA content in the initial feed sample was 11 ng/μL, with the DNA concentration in the retentate product equal to 76 ng/μL; this is considerably smaller than the 11‐fold increase in mAb concentration (from 0.7 to 8 g/L). DNA concentrations in permeate samples collected during SPTFF were quite small, suggesting that a significant fraction of the DNA was removed by adsorption to the membrane (and not transport into the permeate).

At the conclusion of the SPTFF, the mAb solution in the module and feed / recycle line was collected in the feed reservoir, yielding a feed stock of approximately 8 g/L mAb, 76 ng/μL of DNA (9500 ppm), and 48,000 ppm (0.38 mg/mL) of HCP. The Cytiva 100 kDa UF membrane was selected for HPCMP based on its high BSA/IgG diffusive selectivity of 250, which was even greater than the selectivity evaluated for the dialyzers (Table 2). The HPCMP process was operated in single pass mode at a feed flow rate of 0.005 mL/min using PBS as the draw solution with *q*_D_/*q*_F_ = 4. Note that this q_D_/q_F_ ratio indicates that the total buffer usage for the HPCMP process is only 4 times the feed volume, which is significantly less than the buffer usage for both affinity chromatography (accounting for the column equilibration, washing, and elution steps) and typical diafiltration (which uses 5–10 times the feed volume). Grab samples from the retentate outlet remained stable at 8.0 ± 0.2 g/L throughout 48 h of continuous operation (processing >14 mL of the concentrated feed), with a mAb yield of 98% ± 2%. There was also no change in feed‐side pressure drop over the course of the HPCMP experiment.

The SEC analysis of the final product in Figure 7 showed near‐complete elimination of peaks eluting at *t* > 23 min, representing a >99% reduction in low MW species relative to the original feed (shown in Figure 6 – the difference in the absorbance values between the SEC profiles for the retentate in Figure 6 and the feed in Figure 7 is due to the normalization of the absorbance values used in Figure 6 to highlight the HCP removal). This exceptional performance remained stable over 48 h of continuous operation, demonstrating that any adsorptive effects (which likely saturate after a short period of time) had minimal impact on the membrane performance. No evidence of increased high MW species (eluting before the mAb) was observed, consistent with HPCMP's selective action on low‐MW solutes.

**FIGURE 7:**
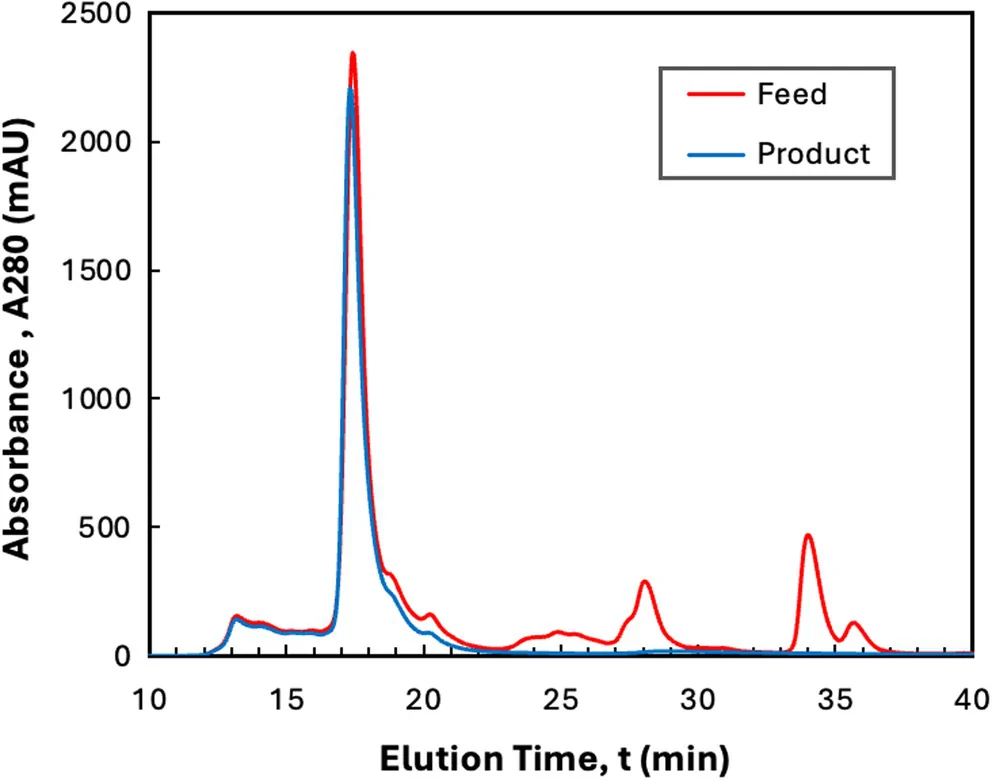
SEC chromatograms of the feed and retentate (product) obtained during the HPCMP experiment after 48 h performed with the 100 kDa Cytiva membrane with the preconditioned CCF.

In addition to SEC analysis, the reduction of host cell protein (HCP) levels was quantified by ELISA, with results summarized in Table 3 at each stage of the purification process. The HCP levels are shown as ppm, evaluated as the ratio of the HCP concentration to the mAb concentration in each sample. The SPTFF step achieved a reduction in the HCP concentration by ~66%. Subsequent processing by HPCMP provided an additional reduction of ~63% relative to the retentate obtained by SPTFF. Cumulatively, the combined SPTFF‐HPCMP process achieved a total HCP reduction of ~92% (mass of HCP relative to mass of mAb) compared to the initial cell culture fluid, from 224,000 to 18,000 ppm. These results are similar to those reported by Minervini et al.^15^ for a combined SPTFF‐HPCMP process performed using the Revaclear 400 for the SPTFF and the 3M dialyzer for the HPCMP.

**TABLE 3:** HCP and DNA removal for the combined SPTFF and HPCMP process using Cytiva 100 kDa UF membrane.

	mAb concentration (g/L)	HCP (ppm)	DNA concentration (ng/μL)
CCF	0.7	224,000	11
SPTFF product	8.0	48,000	76
HPCMP product	8.0	18,000	18

## 4 DISCUSSION

The results presented in this study establish that commercial UF membranes engineered for pressure‐driven separations and already validated for biopharmaceutical manufacturing can be repurposed for highly selective diffusive purifications via HPCMP. This finding is significant because it eliminates a critical barrier to industrial adoption – namely, the need for specialized, non‐validated membrane materials. Although the UF modules showed somewhat poorer selectivity than the dialyzers when using polydisperse dextrans, the selectivity observed with the Cytiva 100 kDa polysulfone membrane for the diffusive separation of BSA and IgG selectivity was >150‐fold, which exceeds that of prototype hemodialysis membranes. This high selectivity arises from a combination of steric effects (inverse relationship between bulk diffusion coefficient and solute size) and hindrance to diffusion in the restricted pores of the membrane, possibly in combination with protein adsorption, which further reduces the rate of diffusive transport through the membrane. Note that the difference in behavior between the model proteins and the dextrans is likely due to differences in physical characteristics (highly globular vs. flexible chains) and differences in solute‐membrane interactions for the proteins and dextrans.

The integrated SPTFF‐HPCMP platform demonstrates substantial purification of a model mAb from crude cell culture fluid with >98% mAb recovery, ~92% HCP reduction, >99% removal of low‐molecular‐weight impurities, and more than a 10‐fold concentration factor. In addition, the performance of the HPCMP process remained nearly constant over 48 h of continuous operation, suggesting that it may be possible to incorporate this technology as part of a fully continuous mAb purification process.

The Process Mass Intensity^25^ (PMI) for the SPTFF‐HPCMP process:

(9) $$\mathrm{PMI}=\frac{\text{Total input mass}\;\left(\mathrm{kg}\right)}{\text{Mass of active product}\;\left(\mathrm{kg}\right)}$$

is PMI = 500 based on the use of *q*_D_/*q*_F_ = 4 (neglecting the small mass associated with the hollow fiber module itself). Budzinski et al.^25^ performed a benchmarking study across multiple biomanufacturing companies with results indicating that typical mAb processes had PMI = 2000–25,000, with the bulk of the PMI arising from the downstream process. The relatively low PMI for the SPTFF‐HPCMP process, coupled with the significant degree of purification, suggests that this platform could serve as an effective initial purification step in a continuous manufacturing process. The relative simplicity of the operation, requiring only standard, off‐the‐shelf membrane modules and peristaltic pumps, with fully steady‐state operation, is conducive to rapid technology transfer and scale‐up (assuming that the flow distribution in the fiber lumens and shell region remains uniform in both small‐ and large‐scale hollow fiber modules). The uniformity of flow in the diffusive mode of operation will need to be demonstrated in large scale modules, although previous results with the dialyzers suggest good performance in modules up to at least 2 m^2^ of membrane area.

Although the SPTFF‐HPCMP process examined in this work was applied directly to the CCF (before any chromatographic purification), this is not the only possible opportunity for implementation of this technology. For example, HPCMP could be employed as a conditioning step after initial capture (e.g., by Protein A chromatography), both to reduce HCP and DNA concentrations while simultaneously performing buffer exchange to optimize the pH and conductivity for subsequent polishing steps. Alternatively, SPTFF and HPCMP could be used for final formulation (in place of traditional batch ultrafiltration / diafiltration), providing concentration, buffer exchange, and additional removal of trace impurities that might remain even after polishing chromatography. The most effective incorporation of this technology within the overall downstream process is beyond the scope of the current work.

Future work shall address several important considerations: (i) the mechanism of the elevated IgG selectivity observed with polysulfone membranes despite their larger pore size warrants mechanistic investigation through techniques such as confocal microscopy (to quantify surface fouling), proteomics (to identify which HCP species are preferentially removed), and size‐fractionated DNA analysis (to clarify nucleic acid removal mechanisms); (ii) process optimization studies examining the impact of buffer ionic strength, pH, and flow rate ratio would establish the robustness and generalizability of the approach; (iii) integration of SPTFF‐HPCMP with downstream polishing steps such as flow‐through membrane chromatography would facilitate the development of a fully continuous mAb manufacturing platform; and *(iv)* application of the technology with more challenging to process mAbs and mAb‐related products, for example, bispecifics.

## 5 CONCLUSIONS

There is growing interest in the development of low‐cost strategies for purification of monoclonal antibody therapeutics, particularly for high volume applications in low‐ and middle‐income countries. Commercial hollow fiber UF membranes, initially designed for pressure‐driven convective separations, can be effectively repurposed for highly selective diffusive purifications via High Performance Countercurrent Membrane Purification. When applied to monoclonal antibody purification from CCF, an integrated SPTFF‐HPCMP platform can achieve high product recovery and significant reductions in process‐related impurities. These results demonstrate the practical feasibility of adopting HPCMP using commercially available membranes to develop scalable, continuous manufacturing platforms for low‐cost biopharmaceutical production. The capacity to leverage existing, regulatory‐approved materials substantially accelerates the path to industrial implementation and should facilitate broader adoption of membrane‐based purification strategies in biotherapeutic manufacturing.

## AUTHOR CONTRIBUTIONS

**Siavash Darvishmanesh:** Investigation; methodology. **Aylin Mohammadzadehmarandi:** Conceptualization; investigation; methodology; formal analysis; writing – original draft; data curation. **Stefano Menegatti:** Formal analysis; supervision; investigation; writing – review and editing. **Nicholas Marchand:** Investigation; methodology. **Ka Zhang:** Formal analysis; investigation. **Jon Petrone:** Investigation; methodology. **Mohammadreza Ahmadimashhadi:** Investigation; data curation; formal analysis. **Andrew L. Zydney:** Conceptualization; methodology; formal analysis; writing – review and editing; funding acquisition; supervision. **Nate Heller:** Data curation; investigation.

## CONFLICT OF INTEREST STATEMENT

JP, NM, and SD are employees of Cytiva, which manufactures some of the hollow fiber modules used in this work.

## Data Availability

The data that support the findings of this study are available from the corresponding author upon reasonable request.
